# Editorial Note: MiR-155 Induction by *F*. *novicida* but Not the Virulent *F*. *tularensis* Results in SHIP Down-Regulation and Enhanced Pro-Inflammatory Cytokine Response

**DOI:** 10.1371/journal.pone.0307496

**Published:** 2024-07-25

**Authors:** The PLOS ONE Editors

Following the publication of this article [1], concerns were raised regarding results presented in Figures 1 and 6. Specifically,

- The Figure 1A IB: SHIP and IB: Actin panels are duplicated as lanes 1–2 of the Figure 6C IB: SHIP and IB: Actin panels
- There appear to be vertical irregularities suggestive of splice lines in the Figure 1B IB: Actin and Figure 1C IB: Actin panels. Similar irregularities were not detected in the corresponding IB: SHIP and IB: SHIP1 panels.

During initial editorial discussions, the corresponding author stated that the Figure 1A and 6C panels are duplicated because these panels intentionally present the same results. The corresponding author provided the original blots underlying the Figure 1A and 6C results, which have been provided in the S1 and S2 Files available with this notice.

However, the authors did not respond to follow-up queries regarding the observed irregularities in the IB: Actin panels of Figure 1B and 1C. Original data underlying the Figure 1B and 1C results were not provided for editorial review. In the absence of the original image data underlying these published panels, the Figure 1B and Figure 1C concerns cannot be fully resolved. Claims based on Figures 1B and 1C should be interpreted with caution; of note, Figure 1C reports the only experiment in the article evaluating SHIP expression in macrophages following *F*.*n*. treatment.

The *PLOS ONE* Editors issue this Editorial Note to notify readers of the unresolved issues with Figures 1B and 1C, and to relay the available data underlying Figures 1A and 6C provided by the corresponding author.

## Supporting information

S1 File Original blots underlying the Figures 1A and 6C IB: SHIP results. (JPG)

S2 File Original blots underlying the Figures 1A and 6C IB: Actin results. (JPG)
