# Supplementary figures and images for: Editorial Note: MiR-155 Induction by F. novicida but Not the Virulent F. tularensis Results in SHIP Down-Regulation and Enhanced Pro-Inflammatory Cytokine Response
Source: PLoS One. 2024 Jul 25;19(7):e0307496. doi: 10.1371/journal.pone.0307496 (PMC11271958; doi:10.1371/journal.pone.0307496)

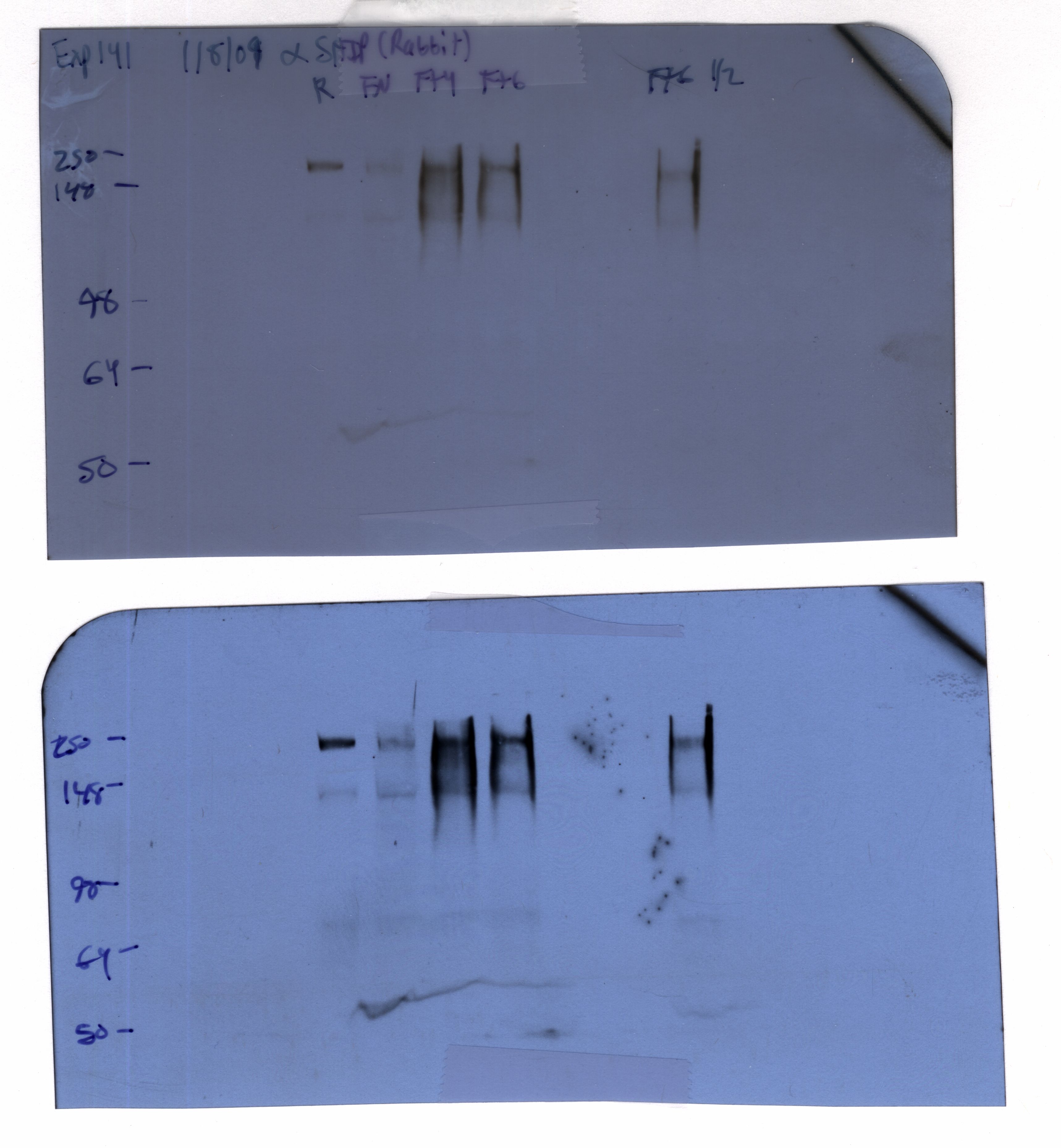

Supplement: S1 File — (JPG) [file pone.0307496.s001.jpg]

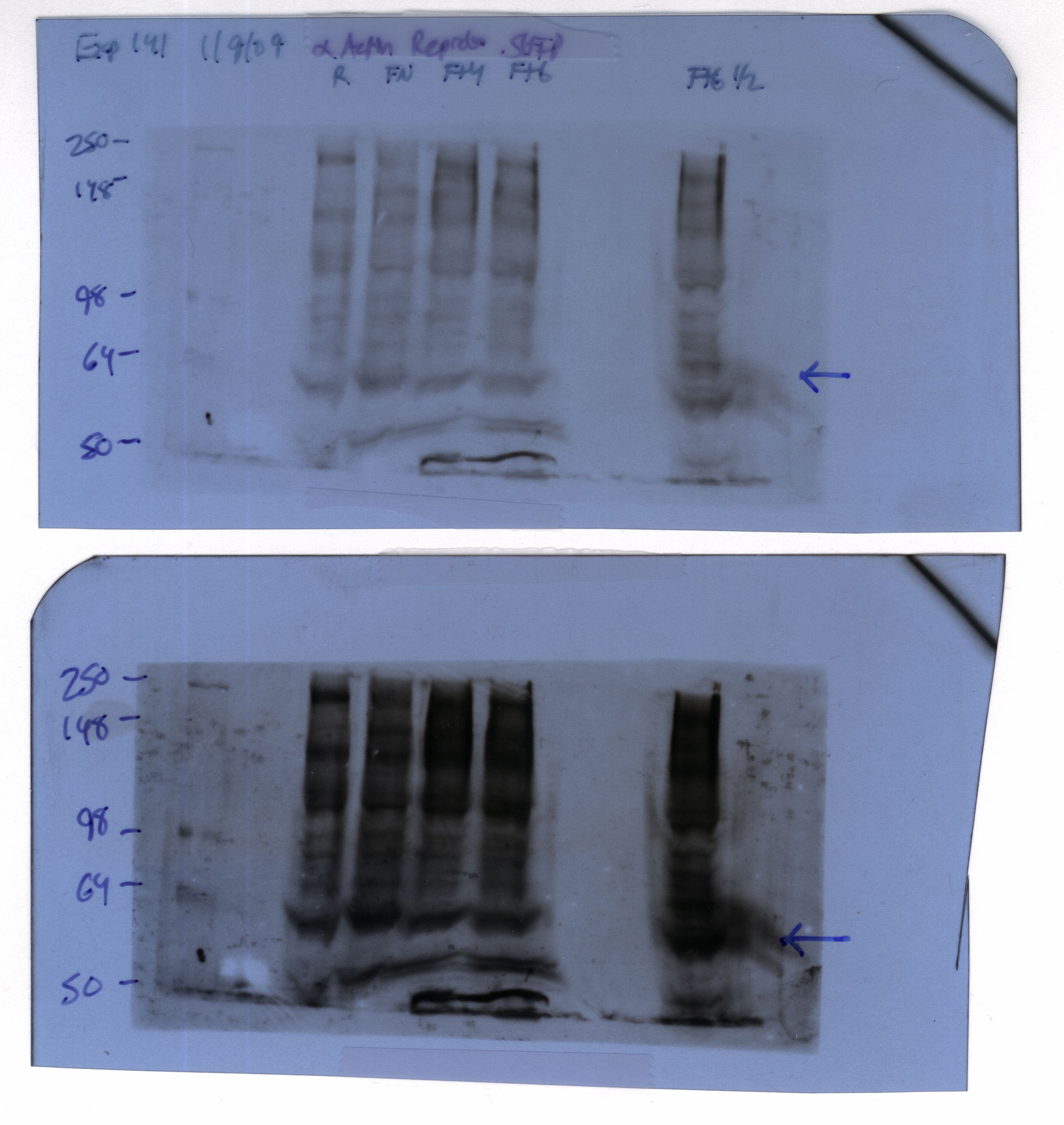

Supplement: S2 File — (JPG) [file pone.0307496.s002.jpg]
